# Zooming in on the initial steps of catalytic NO reduction using metal clusters

**DOI:** 10.1039/d1cp05760j

**Published:** 2022-03-04

**Authors:** Joost M. Bakker, Fumitaka Mafuné

**Affiliations:** Radboud University, Institute for Molecules and Materials, FELIX Laboratory Toernooiveld 7 6525 ED Nijmegen The Netherlands joost.bakker@ru.nl; Department of Basic Science, School of Arts and Sciences, The University of Tokyo, Komaba, Meguro Tokyo 153-8902 Japan mafune@cluster.c.u-tokyo.ac.jp

## Abstract

The study of reactions relevant to heterogeneous catalysis on the surface of well-defined metal clusters with full control over the number of consituent atoms and elemental composition can lead to a detailed insight into the interactions between metal and reactants. We here review experimental and theoretical studies involving the adsorption of NO molecules on mostly rhodium-based clusters under near-thermal conditions in a molecular beam. We show how IR spectrosopic characterization can give information on the binding nature of NO to the clusters for at least the first three NO molecules. The complementary technique of thermal desorption spectrometry reveals at what temperatures multiple NO molecules on the cluster surface desorb or combine to form rhodium oxides followed by N_2_ elimination. Variation of the cluster elemental composition can be a powerful method to identify how the propensity of the critical first step of NO dissociation can be increased. The testing of such concepts with atomic detail can be of great help in guiding the choices in rational catalyst design.

## Abstract 2

The study of reactions relevant to heterogeneous catalysis on metal clusters with full control over the number of constituent atoms and elemental composition can lead to a detailed insight into the interactions governing catalytic functionality.

## Graphical abstract

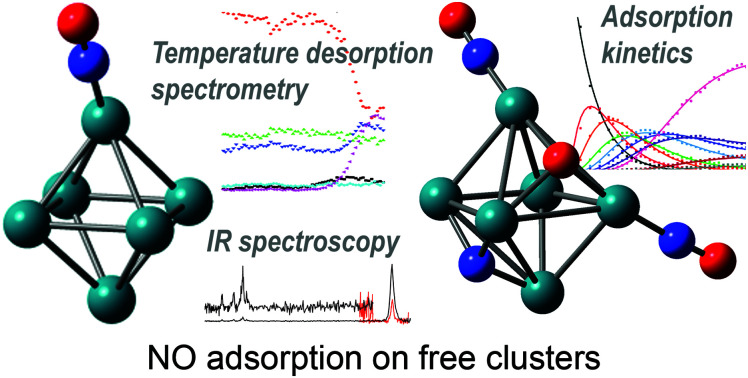

## Introduction

The reduction reaction of nitrogen monoxide (NO),

1 2NO → N_2_ + O_2_

has a negative change in Gibbs free energy and is therefore thermodynamically favorable.^1^ However, the reverse reaction also occurs when fuel is burned at higher temperatures in the presence of N_2_, thus emitting NO to the environment.^2,3^ Once NO has been formed, it remains in the air because reaction (1) has a high activation barrier.^4^ NO molecules in the atmosphere are air pollutants that have a harmful effect on the respiratory systems of animals, and are precursors for tropospheric ozone and aerosols. Therefore, governmental organizations have set stringent laws and regulations to control emissions, such as Euro 5 and Tier 4, and these emission limits have been tightened significantly over the years.^5,6^

To reduce the amount of NO emitted during the burning of fuel, a variety of catalytic systems have been developed. For example, copper-ion-exchanged ZSM-5 zeolite (Cu/ZSM-5) is a well-known catalyst for direct NO decomposition; in this catalyst, copper dimers likely contribute to the catalytic efficiency.^7,8^ Direct NO decomposition is ideal because no other chemicals are required for the chemical reaction. This reaction, however, has never been used practically because it is not efficient in the presence of water.^9^ Further, the exploration of suitable catalysts remains incomplete.

A well-established system used in diesel engines is selective catalytic reduction (SCR), which utilizes ammonia from urea as a reductant in the presence of catalysts,

2 4NO + 4NH_3_ +O_2_ → 4N_2_ + 6H_2_O,

thus forming N_2_ and H_2_O.^10^ However, this system requires NH_3_, and its effectiveness is reduced at lower temperatures.

One of the most practical systems for treating NO emissions from automobile gasoline engines is a three-way catalytic converter (TWC). TWCs mainly contain Pt, Pd, and Rh nanoparticles and effectively reduce the NO, CO, and hydrocarbon (HC) concentration of exhaust gas simultaneously. Here, CO and HC are the reductants for NO, while N_2_, CO_2_, and H_2_O are formed as the final products.^11–20^ Rh is considered to be the main element active in the catalytic chemical reduction of NO. Because of this unique ability, over 80% of the global Rh demand is for TWCs. However, Rh is extremely rare and expensive, and is subject to financial speculations on the commodity markets. As a consequence, the discovery and development of less expensive and more abundant components are crucial.

To find a suitable alternative, it is necessary to understand the unique properties of Rh for the NO reduction reaction.^21–24^ Because the proposed reaction mechanisms involve the dissociation of NO, the adsorption of NO on the Rh(100),^25–30^ Rh(111),^31–40^ and Rh(110)^41–51^ surfaces has been investigated by a number of researchers.

Since the catalyst nanoparticle morphology is typically far from crystalline, it is important to also understand the adsorption of NO onto more irregularly shaped rhodium surfaces. For this, the study of reactions between isolated atomic clusters and NO provides an excellent platform. Clusters can be generated in various sizes, and, using mass-spectrometric techniques, an atomically precise system can be probed. Although cluster geometries and electronic configuration may change significantly upon addition or removal of a single atom, this atomic precision allows for a very detailed study of the adsorption reaction.^52–54^

In this Perspective, we sketch how our and others’ studies of NO adsorption on rhodium clusters can help to understand the geometric and electronic factors that determine whether NO adsorbs molecularly, or whether it dissociates upon adsorption. For this we mainly discuss the experiments we have carried out over the last five years, in which we characterize NO adsorption though IR spectroscopy and Thermal Desorption Spectrometry (TDS), but we will also discuss other related experiments.

## Background

### NO adsorption on crystalline surfaces

Spectroscopic studies have shown that the degree of NO dissociation depends on the face, surface temperature, and coverage. Generally, NO molecules adsorb intactly at low temperatures and dissociate at high temperatures. At low coverages NO dissociates completely, whereas at higher coverages the dissociation is incomplete because dissociation requires an empty neighbor site.^55^

Ho and White found that at 100 K NO chemisorbs on the Rh(100) surface with an initial sticking probability of unity, but the probability decreases linearly until the coverage *θ* reaches saturation at *θ* = 0.65 monolayer (ML).^25^ At 100 K and all coverages, the adsorption of molecular NO is dominant with a small dissociative contribution at low coverages. Ho and White also suggested that the molecular axis of adsorbed NO is tilted with respect to the surface normal. For the desorption of O_2_, N_2_, and NO at 470–495 K at higher coverages (*θ* ≥ 0.25 ML), an intermediate with the N and O atoms directly interacting with Rh atoms and a very weak N–O bond has been proposed.^25^

Based on electron energy loss spectroscopy (EELS), Villarrubia and Ho reported two modes of molecular adsorption: a “lying-down” or highly inclined NO mode (denoted α_1_NO), exclusively formed at *θ* < 0.12 ML, with a characteristic vibrational mode at 919 cm^−1^.^26^ The unusually low stretching frequency of α_1_NO is caused by the weakened N–O bond resulting from the almost side-on adsorption form. In contrast, a vertically adsorbed NO (α_2_NO) at a twofold bridge site was observed at *θ* > 0.13 ML, now with coverage-dependent characteristic vibrational modes at 1581–1678 cm^−1^. The shift is due to reduced d-2π* back donation at higher coverages as a consequence of greater competition for the metal d-electrons. Villarrubia and Ho proposed that α_1_NO is an intermediate in the dissociation of α_2_NO.^26^ Van Tol and Nieuwenhuys confirmed the existence of α_1_NO and α_2_NO by field-emission scanning emission microscopy (FE-SEM).^27^ They also observed that when NO is adsorbed at 300 K, dissociation occurs already at low coverages.^27^ When a Rh(100) surface saturated with NO prepared at 80 K is heated, molecular NO desorption starts slowly at 250 K and reaches a maximum rate around 400 K.^27^ NO dissociation starts around 250 K as soon as vacancies are created by NO desorption, and N_2_ desorption starts at 600 K.

Density functional theory (DFT) calculations indicate that α_1_NO and α_2_NO on Rh(100) are energetically competitive, and that the rotation of the NO molecule between α_1_NO and α_2_NO is facile.^29^ In the same work, the barrier for NO dissociation on Rh(100) surface was reported to be 0.5 eV. The dissociation of NO on Rh(100) surfaces has also been modeled by kinetic Monte Carlo (MC) simulations.^30^

DFT calculations further showed that on Rh(100) the bridge site is the most stable as a site for molecular chemisorption, having a binding energy of −2.68 eV at a coverage of 0.25 ML.^28^ Because the transition state for NO dissociation is characterized by a long N–O distance, the barrier height is governed by the strength of the atom-surface interaction. The relatively low barrier for NO dissociation on Rh(100) is thus due to strong atomic binding of N and O to the surface. Because the chemisorption of N or O is stronger than that of NO, NO dissociation is exothermic at low coverages.

For NO adsorption on Rh(111) there is disagreement regarding the adsorption site. EELS measurements have revealed that the N–O stretching mode shifts from 1480 to 1630 cm^−1^ with increasing NO coverage. Root *et al.* assigned the mode to a single adsorption geometry of two-fold bridge sites at all coverages.^31,32^ In contrast, Kao *et al.* reported different results and an alternative interpretation: the adsorption sites of NO depend strongly on the surface coverage and adsorption temperature. At 0.5 ML coverage, only bridge sites are occupied with an N–O stretching frequency at 1590 cm^−1^. At saturated coverage at 120 K, only disordered bridge site adsorption was observed, whereas, at surface temperatures between 250 and 350 K, NO occupation appeared at on-top sites in addition to bridge sites.^33^ Later, based on static secondary ion mass spectrometry (SSIMS) and temperature programmed desorption (TPD) results, Borg *et al.* reported the presence of two distinct NO adsorption states at 100 K, which they assigned to threefold coordinated NO at low coverages of up to 0.50 ML, and less coordinated, presumably twofold bridged NO at higher coverages.^34^ Further, Kim *et al.* studied the adsorption structure of NO on Rh(111) by X-ray photoelectron diffraction and found that the NO coverage is very close to 0.7 ML and the adlayer consists of molecularly adsorbed NO that does not dissociate. Their structural model consists of NO molecules bound to on-top sites, threefold hollow face centered cubic (fcc), and threefold hollow hexagonal close packed (hcp) sites through the N atom with the molecule axis perpendicular to the surface.^35^ DFT calculations suggested that the threefold hollow site is most stable on Rh(111), having a binding energy of −2.18 eV at a coverage of 0.33 ML.^22^ Later, Yoshinobu *et al.* directly observed the stretching vibrations of NO in on-top and hollow sites at 1816 and 1479 cm^−1^ by infrared reflection adsorption spectroscopy (IRAS).^36^

Currently, consensus can be summarized as follows: the saturation coverage of NO on Rh(111) is approximately 0.67 of the surface atom density. After NO is adsorbed molecularly at 120 K, at low coverages (*θ* < 0.3), NO dissociates completely upon heating around 275–325 K to form N and O. The N atoms combine and desorb between 450 and 650 K, whereas the O atoms desorb as O_2_ at 1000–1310 K. In the medium coverage range (0.25 < *θ*_NO_ < 0.50 ML), some NO dissociates above 300 K until all empty threefold hollow sites are filled. At this point, the dissociation of NO becomes progressively inhibited because of site blocking. Increasing the NO coverage further results in the adsorption of NO on the on-top and threefold hollow sites. At this point, NO dissociation is inhibited until >400 K, where three-fold hollow NO begins to desorb with an estimated desorption barrier of 113 ± 10 kJ mol^−1^. The desorption kinetics of N_2_ is strongly influenced by the presence of coadsorbed oxygen.^31,34,37,38^

At moderate pressures (≤10^−6^ mbar) and temperatures (<275 K), a transition from threefold hollow to on-top bonding was observed by Wallace *et al.* using *in situ* polarization-modulation IRAS. Gas-phase NO is directed toward the on-top position because of the presence of NO decomposition products, particularly chemisorbed atomic O at the hollow sites. Under higher pressure conditions (1 mbar), NO exposure at 300 K results exclusively in on-top NO.^39^

In comparison with the dissociation of NO on Rh(100), that on Rh(111) occurs at higher temperatures, illustrating the higher reactivity of the (100) surface. The largest difference between Rh(111) and Rh(100) is seen in the temperatures of second-order desorption of N_2_ due to the disproportionation reaction NO_ads_ + N_ads_ → O_ads_ + N_2_, from the surface at low coverages (where repulsive interactions between O and N atoms play a minor role): ∼670 K on Rh(111) but ∼810 K on Rh(100).^40^

By comparison with the extended Rh surface, the reactivity of NO with Rh catalysts supported on SiO_2_ is not fully explained by analogy with the Rh surface. Chin and Bell reported that the adsorption of NO occurs primarily in a molecular state and no more than 9% of the adsorbed NO is dissociated at room temperature.^12^

### NO adsorption on rhodium clusters

In general, the structures of gas-phase clusters are not so straightforward to inspect as that of an extended (crystalline) surface. Typically, their structures can be inferred using IR spectroscopy,^56^ trapped ion electron diffraction spectroscopy,^57,58^ or ion-mobility mass spectrometry,^59^ where it should be noted that the latter are only applicable to cationic and anionic clusters. Mackenzie *et al.* investigated the geometric structures of small cationic rhodium clusters (Rh_*n*_^+^, *n* = 6–12) by comparing the experimental infrared multiple-photon dissociation (IR-MPD) spectra and spectra calculated using DFT and found that they form octahedral and tetrahedral motifs.^60,61^ By storing room-temperature Rh_*n*_^+^ (*n* = 7–30) clusters in a Fourier transform ion cyclotron resonance (FT-ICR) type ion trap under ultrahigh-vacuum conditions, ensuring that only a limited number of collision events occur, they observed chemical reactions with multiple NO molecules and analyzed the product ions using mass-spectrometry. For low NO concentrations, they found products involving the simple adsorption of NO, such as [Rh_*n*_,N,O]^+^. The bracket notation is used to indicate the fate of the NO molecule after adsorption is unknown. At higher NO concentrations, key intermediate species such as [Rh_*n*_,2O]^+^ and [Rh_*n*_,4O]^+^ were observed. Such species were interpreted to result from the dissociative chemisorption of multiple NO molecules in the early stages of the reaction, followed by the desorption of N_2_ and the formation of rhodium oxides.^62–64^ For *n* < 13, this process continues until a critical size-dependent oxide cluster is produced, after which further NO molecules are adsorbed without subsequent N_2_ loss. In contrast, larger rhodium clusters such as Rh_13_^+^ and Rh_*n*_^+^ (*n* > 16) adsorb NO until a saturation is reached, without any indication of N_2_ desorption. These observations can be explained in terms of energy and heat capacity: the energy gained upon NO adsorption on a large cluster is distributed over a correspondingly larger number of internal degrees of freedom, resulting in only a small increase in the effective cluster temperature. In contrast, when adsorption occurs on a smaller cluster, the same energy is dissipated into fewer vibrational modes. Thus, if NO adsorbs onto a cluster with already one molecularly bound NO, it is likely that the barrier towards dissociation can be overcome. Further adsorptions may then result in the formation and desorption of N_2_.

In similar experiments albeit at slightly higher pressures, Ichihashi *et al.* investigated the reactions of isolated alloy clusters, Rh_*n*_X^+^ (X = Al, V, and Co) with NO molecules and reported NO decomposition *via* the observation of Rh_*n*_XO_2_^+^ formation.^65^ In particular, they found that doping by vanadium is most effective for NO decomposition. They rationalized the decomposition in terms of the formation of a stable dopant metal–oxygen bond and an accompanying increase in adsorption energy, thereby enhancing NO decomposition on the small Rh_*n*_X^+^ clusters studied. Sakaki *et al.* theoretically investigated the reactivity of NO molecules on M_13_ and M_55_ clusters (M = Ru, Rh, Pd, and Ag) using DFT.^66^ They predicted that NO dissociative adsorption occurs on M = Ru and Rh because the high-energy 4d band is favorable for the formation of strong M–N and M–O bonds, which leads to NO dissociative adsorption.

Despite these findings, it remains necessary to investigate (1) the adsorption forms of NO molecules on clusters in the primary step of the complex reaction. Although the mass-spectrometric findings described above are consistent with NO cleavage only after a second NO adsorbing, already the first adsorption may be dissociative. (2) What are the key factors that determine whether NO dissociates? (3) Can NO reduction on a cluster also proceed at room temperature in thermal equilibrium? (4) If not, from which temperature does this occur?

To answer these questions, we have studied products resulting from reacting rhodium clusters with NO under multi-collision conditions. These reactions take place in flow-tube type reaction cells with helium as thermalization agent, at pressures still well below 1 atmosphere, but with collisions with helium dominant. Although full thermalization is difficult to ascertain, the energy released upon adsorption is likely largely dissipated in collisions with the helium, as will be evident below. To diagnose the reaction products, we studied their structure and thermal stability. For the former we used IR-MPD spectroscopy using the FELIX IR free-electron laser. This technique has been successfully used to infer the structure of cluster–molecule complexes.^67–72^ For the thermal stability, we used thermal desorption spectrometry (TDS) to analyze until what temperatures cluster-NO complexes survive, from which the binding energies can be inferred.

## Methods

### Rhodium cluster production and reaction under thermalized conditions

**Fig. 1:**
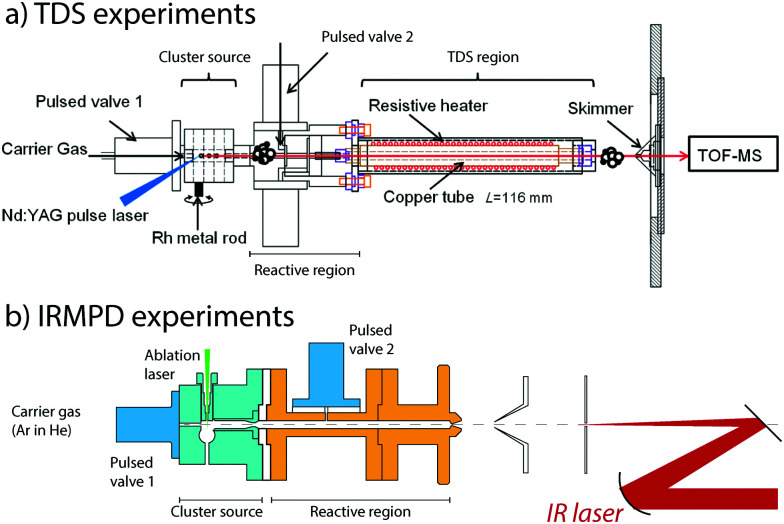
Experimental configurations of temperature programmed desorption (TDS, panel a) and IR multiple-photon dissociation (IRMPD) spectroscopy (panel b) experiments.

To generate clusters with the highest versatility and low material consumption, laser ablation, which was pioneered in the early the 1980s by the Smalley and Bondybey groups,^73,74^ is still a very widely used method. For design considerations, we refer the interested reader to an excellent review.^75^ In our laboratories, we employ a rotating rod design combined with a several centimeter-long cylindrical chamber in which cluster growth occurs. Both sources are quite similar and are detailed in Fig. 1. Solid rods of either a pure metal or an alloy are rotated at revolution periods on the order of seconds to tens of seconds and are irradiated by the loosely focused output (10–50 mJ pulse^−1^, approximately 0.2 mm diameter spot size for rhodium) of the second harmonic (532 nm) of 10 or 20 Hz Nd:YAG lasers. Helium carrier gas is pulsed into the ablation and cluster growth channel employing a general valve, having pulse lengths of 200–300 μs and backing pressures of approximately 7 bar. For IR-MPD spectroscopy experiments, helium is admixed with a low concentration of Ar at typical concentrations of 0.1–1%. For TDS experiments on oxidized clusters, O_2_ is mixed using a mass flow and pressure controller.

The nascent distribution of clusters is entrained by the carrier gas pulse, and several centimeters downstream reacted with NO molecules. These are injected through a second pulsed valve, either pure or diluted in helium. The pulse of NO should not be released too soon, because the reactant may otherwise reach the ablation zone, where reactions under quasi-thermal conditions mediated by collisions with the helium carrier gas only cannot be guaranteed. The mixture is allowed to react while continuing its journey along the flow tube-like channel, until the channel widens, either for expansion into vacuum for the IR-MPD spectroscopic experiments or to subject the mixture to elevated temperatures in the TDS zone. Conditions in the reaction zone are varied, typically at room temperature, to yield populations of M_*n*_^+^-(NO)_*m*_, with *m* optimized for the desired type of experiment. For TDS experiments, a larger *m* is typically desirable, because the temperatures the product species are subject to will span regimes where physisorption and chemisorption are readily discriminated.

In thermal desorption spectrometry experiments, the clusters travel down a 116 mm long extension of the reaction zone formed by a copper tube of larger diameter, which reduces the number of reactive collisions.^76–85^ Crucially, this tube is thermally isolated from the reaction zone and can thus be heated without significantly changing the temperatures of the source and the subsequent reaction zone. In addition, the helium carrier gas cannot escape and forms a thermal link with the heated tube wall, thus ensuring that the reaction products thermalize. Heating is achieved using a thermally anchored but electrically isolated tungsten wire, wrapped around the copper tube. Achievable temperatures range from room temperature to 1000 K, as monitored by thermocouples. The residence time of the cluster ions and the helium number density in the extension tube have been estimated to be approximately 100 μs and 10^17^ cm^−3^, respectively. Hence, the thermal equilibrium of the clusters was achieved. Upon exiting the TDS zone, the gas mixture expands into vacuum, is collimated by a 2 mm diameter skimmer, and is sampled in a reflectron time-of-flight mass spectrometer (RETOF-MS).

In spectroscopy experiments, the reactive mixture consisting of helium, clusters, and NO is expanded into a vacuum, thus forming a molecular beam. The beam is collimated by a skimmer and a 1 mm diameter aperture and is then irradiated by a counter-propagating IR laser. Laser focus and molecular beam collimation are chosen as a compromise of the number of clusters detected and a maximum in the IR fluence observed by the clusters. In practice, the laser focus coincides with the aperture by employing a 450 mm radius of curvature spherical mirror. After irradiation by a typically 6–10 μs macropulse of the FELIX free-electron laser,^86^ all ions travel approximately 50 μs before reaching the source region of a RETOF-MS where they are analyzed.

Irradiation at frequencies coinciding with an IR-active vibrational mode of a cluster–molecule complex can lead to the sequential absorption of several IR photons. The energy absorbed is redistributed through intramolecular vibrational redistribution, leading to the heating of the complexes and their subsequent fragmentation.^87^ Because the fragmentation energy threshold for a cluster–molecule complex is typically on the order of 0.5–1 eV, and the IR photon energy significantly less (0.1 eV at 800 cm^−1^), observation of fragmentation for the weakest IR bands is often not possible. To observe the weaker bands for all species, we have employed the messenger technique first developed by Lee and co-workers,^88^ and later pioneered for metal clusters by Knickelbein for UV-visible spectroscopy,^89^ and by Fielicke for IR spectroscopy.^56^ In this process, a small fraction of Ar is admixed with the carrier gas, leading to the formation of M_*n*_(NO)_*m*_^+^Ar_*p*_ species. IR absorption is then observed on the loss of the weakly bound Ar atom(s). Because the formation of an Ar-tagged complex requires at least some degree of collisional cooling, the study of M_*n*_(NO)_*m*_^+^ reaction products is restricted to the cooler part of the product distribution. Nevertheless, we have not observed significant deviations between the spectroscopic and TDS results.

In IR-MPD spectroscopy, mass channel intensities are conventionally converted into (relative) molecular absorption intensities, which eliminates the production efficiency. In a typical treatment, the fragmentation efficiency is assumed to be single-photon-like, and the fragment and parent populations *F* and *P* are related by

3 *P*(*ν*) = *P*_0_e^−*σ*(*ν*)*f*(*ν*)^,

4 *F*(*ν*) = *P*_0_(1 − e^−*σ*(*ν*)*f*(*ν*)^),

where *P*_0_ is the parent population prior to irradiation, *σ*(*ν*) is the IR absorption cross-section, and *f*(*ν*) is the photon flux density per macropulse at frequency *ν*. The fragmentation yield *Y*_F_(*ν*) is retrieved as

5 *Y*_F_(*ν*) = −ln(*P*(*ν*)/*P*_0_) = −ln(*P*(*ν*)/(*P*(*ν*) + *F*(*ν*))),

which for *F*(*ν*) ≪ *P*(*ν*) reduces to *F*(*ν*)/*P*(*ν*). This reduction eliminates shot-to-shot fluctuations and long-term drift in the parent ion production. In most studies evaluating cluster distributions, IR irradiation drives the back reaction, *i.e.*, the reaction where the adsorbed Ar atom desorbs again, for instance,

6 M_*n*_^+^(NO)_*m*_Ar_*p*_ + *n*h*ν* → M_*n*_^+^(NO)_*m*_Ar_*p*−1_ + Ar.

Because the fragment channel in cluster distributions is typically not empty and, worse, its population prior to irradiation is often significantly larger than that of the parent, production fluctuations rule out the use of eqn (5). Therefore, IR-MPD spectroscopy experiments are typically run at twice the IR laser repetition rate, allowing for the recording of reference mass spectra between two IR laser pulses. Under such conditions, the depletion yield *Y*_D_(*ν*) can be obtained using the depletion *D*(*ν*):

7 *Y*_D_(*ν*) = −ln(*D*(*ν*)) = −ln(*P*(*ν*)/*P*_ref_),

where *P*_ref_, instead of *P*_0_, is the parent population in the reference mass spectra. This treatment reduces long-term drift in cluster production, but not shot-to-shot fluctuations. Because the latter are a significant source of errors, we can eliminate them by calculating the branching ratio of mass channel intensities *I* of all products with Ar attached to those of all products:8
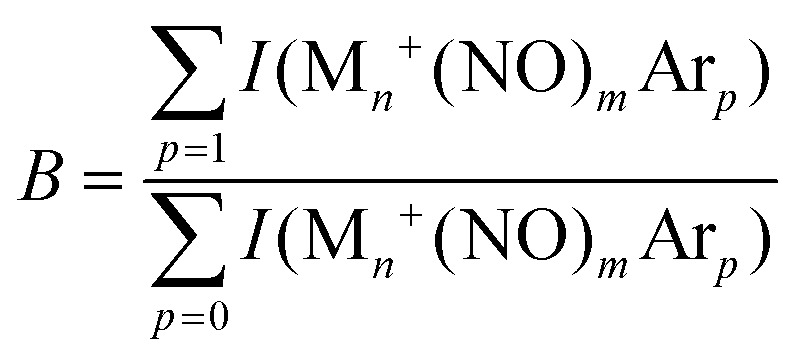
and calculating the yield from

9 *Y*_*B*_(*ν*) = −ln(*B*(*ν*)/*B*_ref_).

Here, *B*_ref_ is the branching ratio in the reference mass spectra. In this treatment, it is implicitly assumed that (i) the Ar complex formation is constant, and (ii) the loss of Ar from M_*n*_(NO)_*m*_^+^Ar_*p*_ always follows equation (3), and no direct loss of two Ar atoms takes place.

**Fig. 2:**
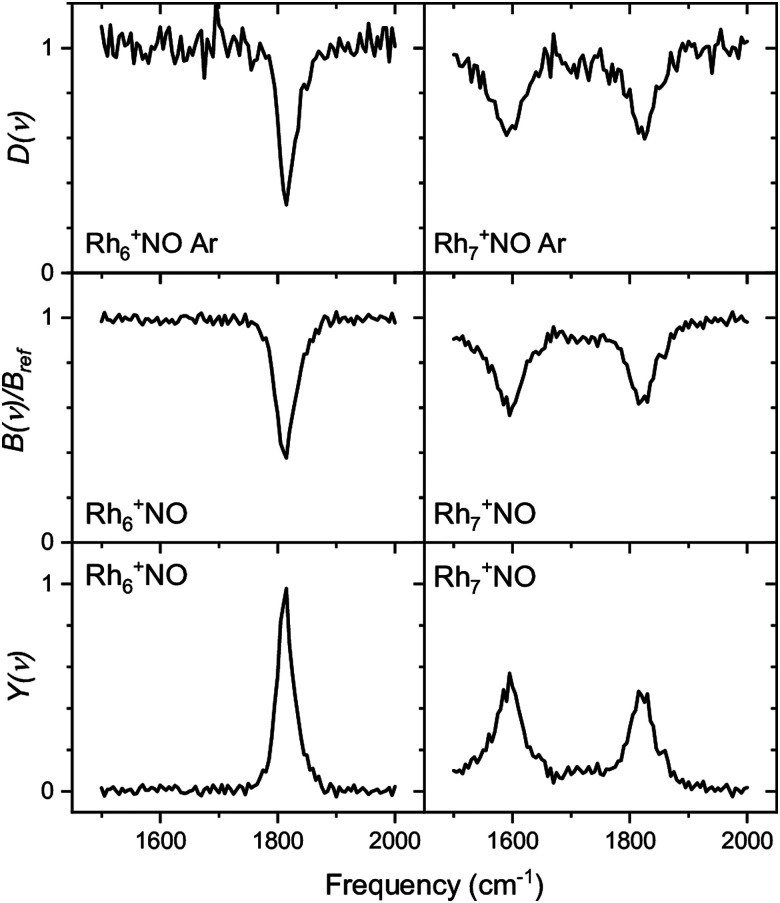
Comparison between depletion *D*(*ν*) for Rh_*n*_^+^NO·Ar (top rows), branching ratio depletion *B*(*ν*)/*B*_ref_ (middle row), and photofragmentation yield *Y*_*B*_(*ν*) for Rh_*n*_^+^NO·Ar_*m*_ (*m* = 1–3) for *n* = 6 and 7. Data from ref. 91.

Whereas the yield *Y*(*ν*) is the most straightforward observable linking a spectrum to the absorption cross-section (the linearity of *Y* with laser pulse energy has been demonstrated in various studies^90^), the depletion *D*(*ν*) can provide information about the potential coexistence of isomeric populations. Examples of the direct depletion of a single mass channel and the branching ratio of all Ar-tagged Rh_6_^+^NO and Rh_7_^+^NO are displayed in Fig. 2.^91^ The top panels show the direct depletion of Rh_6_^+^NO·Ar and Rh_7_^+^NO·Ar in the 1500–2000 cm^−1^ region. The observation of the single band for Rh_6_^+^NO·Ar at 1820 cm^−1^ depleting to approximately 35% indicates a large majority of species of a single isomer (or multiple isomers with the same NO η^1^/on-top adsorption configuration). In contrast, the presence of two depletion bands for Rh_7_^+^NO·Ar at 1580 and 1820 cm^−1^, both depleted to approximately 60%, suggests the co-existence of (at least) two isomeric populations (with NO in η^1^, and in η^2^/bridge configuration). The depletion of the sum of Ar-tagged species normalized to their branching ratio without irradiation gives a spectrum with significantly improved signal-to-noise ratio but no significant shifts, suggesting that this treatment to obtain a spectrum for singly Ar-tagged species is indeed valid, and the direct loss of two or more Ar is insignificant.

### DFT calculations

The absence or presence of bands indicative for NO stretching vibrations and their frequencies can thus already be diagnostic for the nature of adsorption. Similarly, the frequencies for vibrations involving adsorption of atomic N and O are also diagnostic for their local structures. However, even knowing local structure, it can be difficult to reconstruct a complete structure of the cluster-NO system, even for small clusters such as Rh_2_Ta^+^NO. Moreover, to understand the adsorption process, knowing the product structure alone is not sufficient: understanding the energetics and kinetics after initial adsorption is also important.

To know the complete structure of the reaction product, comparison of an IR-MPD spectrum with the vibrational spectra calculated for stable trial geometries is a well-established method. However, as parameter space is already large for relatively small clusters, the computational efforts require some initial screening: an initial optimization of a wide range of structures at lower computational costs is followed by re-optimization to obtain more refined structures and energetics.

There is continuing discussion on how to perform the initial global search without missing stable geometries and which methods and basis sets should be adopted while maintaining computational speed and accuracy. For the initial global search, it is necessary to sample the potential energy landscape of the cluster comprehensively. A wide variety of methods are available, including basin-hopping methods,^92^ genetic algorithms,^93,94^ and random positioning.^95,96^

**Fig. 3:**
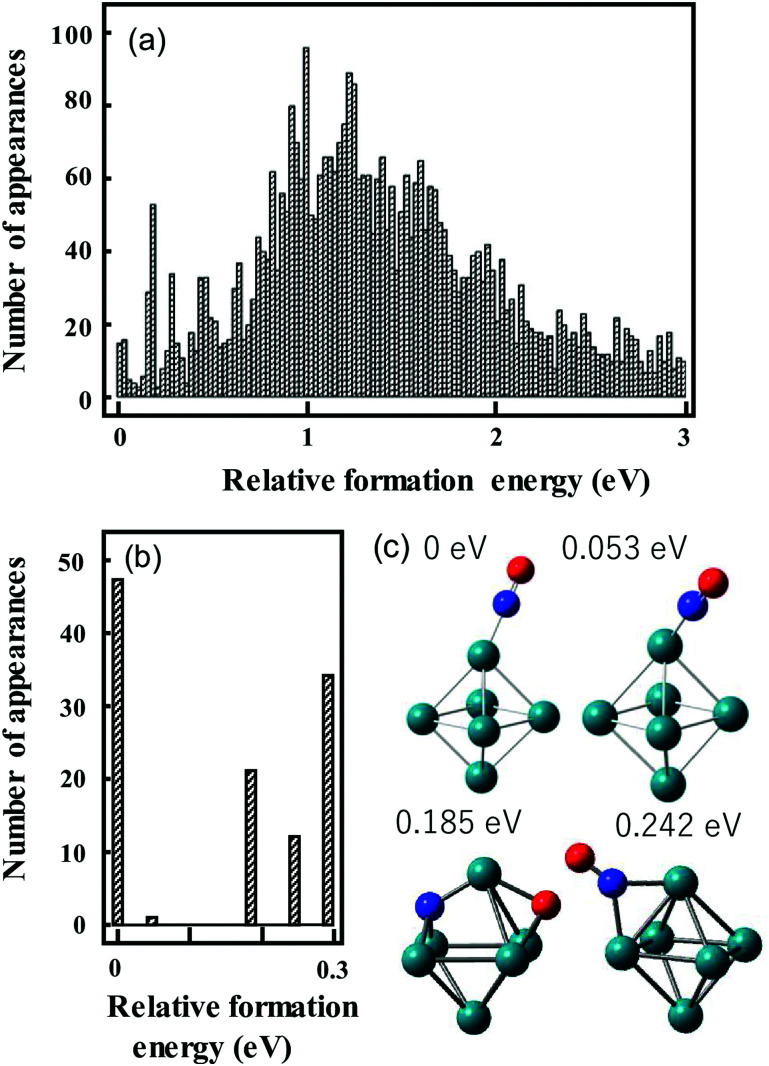
Histograms of relative energies of Rh_6_^+^NO isomers calculated at the B3LYP/LANL2DZ (a) and B3LYP/sdd (b) levels of theory. For (b) all geometries in the [0,0.5 eV] interval in (a) were reoptimized. (c) Geometries of the four lowest-energy isomers in (b). Panel (a) reprinted with permission from ref. 97. Copyright (2021) Elsevier.

We have used the following procedure. For the initial global structure optimizations, low-energy geometries are explored using small basis sets such as the Los Alamos National Laboratory 2-Double-ζ (LANL2DZ) basis sets with >1000 initial geometries in which atoms are randomly positioned in a 3D space on the condition that given two atoms are located within a reasonable distance. The spin multiplicity for each structure is changed to consider all conceivable spin states. Calculations are repeated until the same low-energy geometries are obtained from multiple different initial geometries. Therefore, many more initial geometries are calculated for metal clusters that have different stable geometries with a similar formation energy. Geometries with energies up to *ca.* 2 eV higher than that of the most stable structure are re-optimized using larger basis sets such as the Stuttgart/Dresden (SDD) basis sets. An illustration of this is shown in Fig. 3a, which shows the energy distribution of Rh_6_NO^+^ structures optimized using the smaller basis set.^97^ Calculations with a larger basis set for the subset of lower energy isomers refines this picture in Fig. 3b. Here, we find that using the larger basis set for all structures found below a certain energy threshold, typically 0.5 eV, they collapse into a limited number of structures that can be categorized in *families* of structures with very similar geometric characteristics and spectral fingerprints. From each family, we take the lowest energy structure as representative, with different spin multiplicities (2*S* + 1 = 5, 7, 9).

Once structures have been optimized using the larger basis set, vibrational spectra are constructed by calculating harmonic frequencies and IR intensities for the most stable geometries. We typically do not scale our calculated frequencies, a common practice to correct for anharmonicity, because we have found scaling of metal–metal vibrational frequencies can require very different factors than for instance the NO stretching vibration.

The choice of functional is also worth some consideration. In previous work on open-shell clusters (as Rh_*n*_^+^ clusters are) we have evaluated two exchange correlation functionals:^98^ the generalized gradient approximation (GGA) Perdew–Burke–Ernzerhof (PBE)^99^ functional and the revised meta-generalized gradient approximation (*meta*-GGA) Tao–Perdew–Staroverov–Scuseria (revTPSS)^100,101^ functional. Neither of these functionals clearly outperforms the other in predicting the IR spectra, and relatively low scaling factors of 0.82 (PBE) and 0.8 (revTPSS) are required to model the spectra, which of course were only showing metal–metal vibrations. Walsh and co-workers used a hybrid PBE variation (PBE1)^102^ for the description of cationic Rh_*n*_^+^·Ar clusters, which appeared to match slightly better.^61^ For the works described here, where NO is adsorbed onto Rh_*n*_^+^ clusters, we made a different choice. Hu *et al.* found that Becke's three-parameter hybrid density functional with the Lee–Yang–Parr correlation functional (B3LYP) provided accurate predictions of Ta–N, N–N, C–H, and N–H bond dissociation energies for Ta_3_N_2_^−^.^103^ We found the B3LYP functional to be quite accurate in predicting metal–N and metal–O vibrations.

## Results and discussion

### Quantifying the adsorption reaction rate of NO onto clusters

**Fig. 4:**
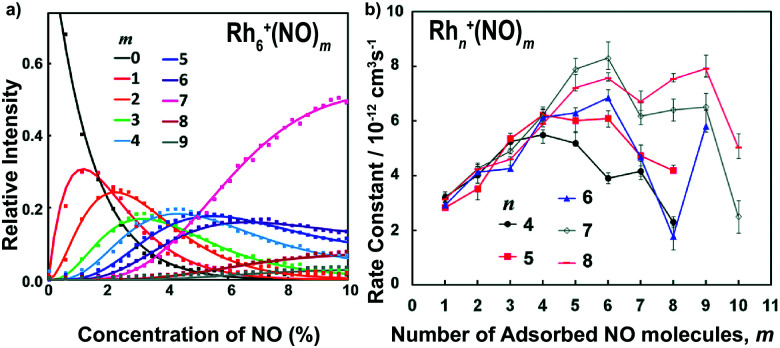
(a) Rh_6_^+^(NO)_*m*_ product formation as a function of NO concentration at room temperature; (b) pseudo-first order rate constants for the reaction Rh_*n*_^+^(NO)_*m*_ + NO → Rh_*n*_^+^(NO)_*m*+1_. Reprinted with permission from ref. 104. Copyright (2015) American Chemical Society.

The adsorption of NO molecules onto clusters is initially investigated by mass-spectrometric evaluation of the product distribution of M_*n*_^+^(NO)_*m*_ as a function of NO concentration. To achieve this, NO gas was diluted in helium and pulsed in *via* a second pulsed valve at a constant stagnation pressure with varying NO partial pressure. In Fig. 4a, the resulting product intensities for Rh_6_^+^(NO)_*m*_ are given at room temperature as the ratio of each product to the sum of products.^104^ The adsorption of up to nine NO molecules on Rh_6_^+^ is observed. The gradual shift of the distribution to larger *m* is indicative of sequential uptake. Note the large abundance of Rh_6_^+^(NO)_7_ at high NO concentrations, suggesting its potential higher stability than species with *m* > 7. The solid curves result from fitting the populations assuming a pseudo-first-order reaction model for sequential coupled reactions, Rh_6_^+^(NO)_*m*_ + NO → Rh_6_^+^(NO)_*m*+1_. The obtained rate constants are shown in Fig. 4b. Here, it becomes clear that the uptake of NO by Rh_6_^+^(NO)_7_ is slower than that by Rh_6_^+^(NO)_6_. The reduced uptake rate for *m ≥* 7 could be rationalized by assuming that each Rh atom in the octahedral Rh_6_^+^, whose structure has been confirmed by spectroscopy,^61^ is occupied, and that subsequent adsorption is a second solvation shell, where the binding is more dispersive in nature, and the charge is diluted with every successive NO molecule, thus decreasing the reaction rate constant. Similar observations can be made for other Rh_*n*_^+^ cluster sizes with reaction rate constants typically peaking at *m* = *n* followed by a rapid decline, although the decline appears less pronounced for larger *n*.

### Vibrational spectroscopy

The analysis of vibrational bands allows us to determine the geometrical structures of species of interest. For molecular NO adsorbed on extended Rh surfaces, NO stretching vibrations are observed at 1544–1643, 1644–1690, and 1800–1850 cm^−1^ when molecular NO adsorbs on a hollow site, a bridge site, and an on-top site, respectively, as listed in Table 1.^105^ If NO is dissociatively bound to the surface, the Rh–O and Rh–N vibrations are found in different spectral ranges. Thus, a vibrational band appearing in a specific wavenumber range is diagnostic for both adsorption form and site.

**Table 1:** Vibrational wavenumbers of species adsorbed on different sites of an extended Rh surface in cm^−1^

Site	NO^a^	O^b^	N
On-top	1800–1850	601, 619, 636	310^c^, 440^d^
Bridge	1644–1690	583, 610	
Hollow	1544–1643	359, 584, 665	

a From Loffreda *et al.*^105^

b Vibration perpendicular to the surface.^106^

c Adsorption site not specified.^107^

d Adsorption site not specified.^44^

**Fig. 5:**
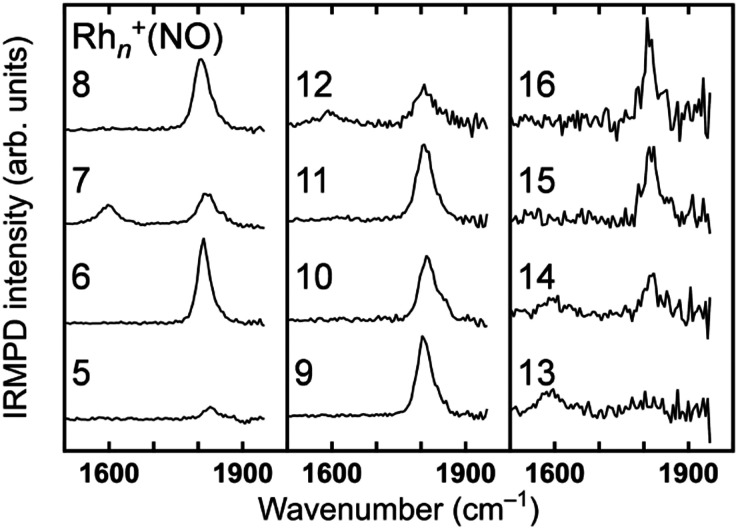
IRMPD spectra of Rh_*n*_^+^NO (*n* = 5–16) between 1400 and 2000 cm^−1^. Data from ref. 91.

**Fig. 6:**
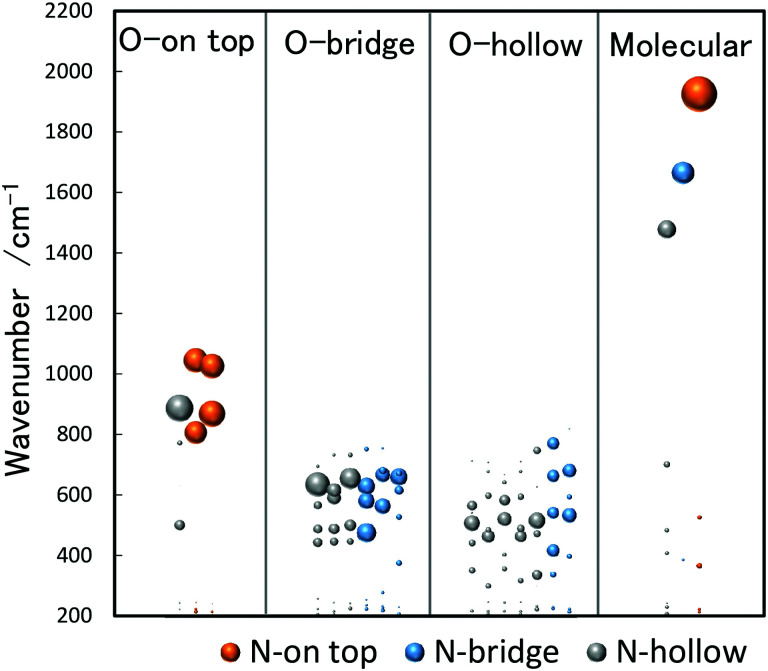
DFT calculated vibrational wavenumbers (unscaled) for different stable structures of Rh_6_^+^NO. The size of each bubble shows the IR spectral intensity of the band. The intensities of the NO stretching vibrations have been reduced by a factor of 10.

The stretch vibrations of molecular NO adsorbed on gas-phase Rh clusters can also be discussed based on the data for the extended Rh surface. Fig. 5 shows the IR-MPD spectra of Rh_*n*_^+^NO (*n* = 5–16) in the 1500–2000 cm^−1^ spectral range. All bands observed here are readily attributed to the NO stretch vibrations,^91^ and NO is thus molecularly adsorbed on cationic Rh clusters. For Rh_7_^+^NO, bands were simultaneously detected at 1600 and 1810 cm^−1^ which following the guide lines in Table 1 should be indicative for hollow and on-top adsorption. However, our calculations indicate differently. Fig. 6 shows a plot of the calculated vibrational wavenumbers for different cluster geometries Rh_6_^+^NO, categorized by the nature of NO adsorption and site. Clusters with molecular NO typically have the NO stretching vibrations around 1800 cm^−1^ if the adsorption site is on-top. For bridging sites, this has shifted to 1600 cm^−1^, and for hollow sites even to 1400 cm^−1^. Thus, the 1600 cm^−1^ band for NO adsorbed on a clusters is more likely due to NO adsorbed on a Rh cluster bridge site. The calculations simultaneously find that hollow-site adsorption is energetically unfavorable for these systems. Thus, the use of such assignment “rules” when translating 1 : 1 from extended surfaces require care. Note that the results in Fig. 5 do not rule out the possibility of a coexistence of clusters with dissociative NO. Fig. 6 further shows that for clusters with molecularly bound NO, no spectral intensity between 700–1300 cm^−1^ can be expected, and that the spectral intensity of bands in the 300–800 cm^−1^ range is substantially lower.

Conversely, the absence of NO stretching bands while there are bands indicative for separate N and O atoms bound to the surface immediately indicates dissociative adsorption. For clusters with dissociatively adsorbed NO, the clusters with the O atom on the on-top site tend to have the N atom on the on-top or hollow site, which are characterized by vibrations at 800–1100 cm^−1^. The clusters with the O atom on the bridge or hollow site tend to have the N atom on the bridge or hollow site, which are characterized by vibrations at 300–800 cm^−1^.

**Fig. 7:**
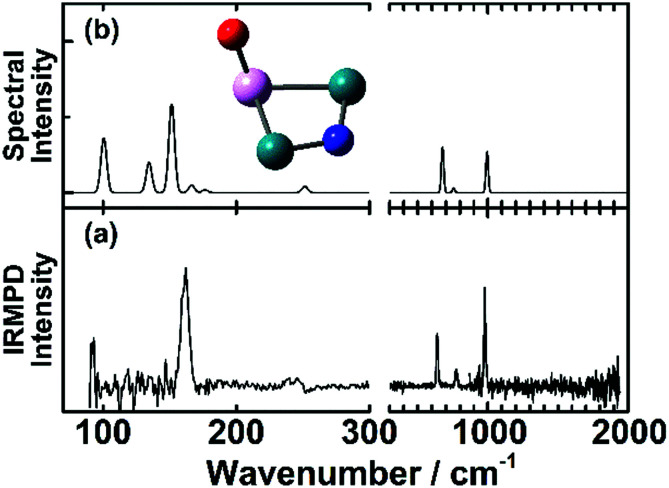
(a) IR-MPD spectrum of Rh_2_Ta^+^NO. (b) DFT calculated spectrum of the most stable Rh_2_Ta^+^NO isomer. Green, pink, blue and red spheres represent Rh, Ta, N, and O atoms, respectively. Experimental data from ref. 108.

An example for dissociative adsorption is shown in Fig. 7, where the IR-MPD spectrum of Rh_2_Ta^+^NO exhibits clear bands at 638, 775, and 978 cm^−1^, but none above 1500 cm^−1^.^108^ The lack of the latter is already indicative for dissociative adsorption, but it is confirmed by the three bands between 500 and 1000 cm^−1^, a spectral region where one expects metal–O and metal–N vibrations. In this specific case, the comparison between theory and experiment allows to assign the spectrum to the cluster shown, with O bound to the Ta, and N on a Rh–Rh bridge site. The band at 978 cm^−1^ is assigned to the Ta–O stretching vibration, and the bands at 638 and 775 cm^−1^ to Rh–N–Rh bending modes with the N atom moving perpendicular and parallel to the Rh–Rh bond, respectively.

### Structure of the reaction product(s)

Apart from the diagnostic bands involving molecular NO and metal–N or metal–O vibrations, the IR spectra of the reaction products yield more bands that can serve to structurally characterize a product, or products if isomeric structures are formed. Because parameter space and isomeric complexity increase with the number of NO molecules adsorbed, spectroscopic work was limited to species with up to three NO molecules.

**Fig. 8:**
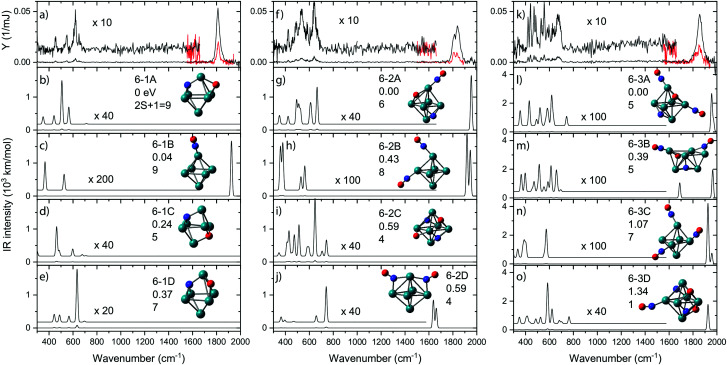
IR-MPD spectra for Rh_6_^+^(NO)_*m*_·Ar, (panels a, f, k) recorded at full (black traces) and reduced (red) IR laser power, accompanied by unscaled harmonic spectra calculated at the B3LYP/sdd/aug-cc-pVDZ level. The associated cluster structures are accompanied by the energy relative to the most stable species, and spin multiplicity. Rh, N, and O atoms are depicted using green, blue, and red spheres, respectively. Data from ref. 91 and 109.

In Fig. 8, IR-MPD spectra are shown for Rh_6_^+^(NO)_*m*_ for *m* = 1–3 over the full 300–2000 cm^−1^ spectral range probed. In each graph, the experimental IR spectrum is shown in the top panel. All three spectra exhibit intense bands at wavenumbers characteristic for intact NO. Because the IR absorption cross-section for NO stretching vibrations is typically two orders of magnitude larger than that for other vibrational modes, the spectrum was also recorded in the 1500–2000 cm^−1^ range using a significantly attenuated IR laser beam (shown in red). All bands observed for the Rh_6_^+^ systems in the 1500–2000 cm^−1^ range (Fig. 8) can be assigned to on-top adsorption (*cf.*Fig. 6). In the calculated spectra for the most stable isomers (panels b–e, g–j, and l–o) all but two structures with intact NO exhibit only on-top adsorption; only structures 6-2D and 6-3B have one or more NO in bridge configuration. All observed bands occurred above 1800 cm^−1^; thus, an easy conclusion would be that all NO adsorbs at on-top sites. Of course, it is not that simple. In the experimental spectrum of Rh_6_^+^(NO), the prominent band at 1815 cm^−1^ is accompanied by weaker bands below 700 cm^−1^ (Fig. 8).^91^ By simply counting the observed resonances it is easy to see that the experimental spectrum contains contributions from several isomers. Although the NO molecule is concluded to mainly adsorb in a molecular form, it is estimated that 2–3.5% of NO adsorbs dissociatively on Rh_6_^+^. This estimate is made by assuming that the relative strengths of the observed bands scale linearly with the calculated IR intensity of each band and the relative population of an isomer. A maximum of the (summed) relative population of isomers with dissociatively adsorbed NO is formed by the maximum population left for bands diagnostic for molecular NO, which for Rh_6_^+^NO amounts to ∼35% as can be seen in Fig. 2.

Rh_6_^+^ preferentially forms an octahedron on which the NO molecule is molecularly adsorbed *via* the N atom on an on-top site (isomer 6B). Adsorption on bridge (+0.41 eV) or hollow (+0.42 eV) sites are less favorable. For dissociative adsorption, N and O adsorb on hollow sites (6-1A) or N on a hollow and O on a bridge site (6-1D, +0.37 eV). Other geometries with N and O adsorbing on different hollow sites are also stable (6-1C, +0.24 eV) but are not observed spectroscopically.

**Fig. 9:**
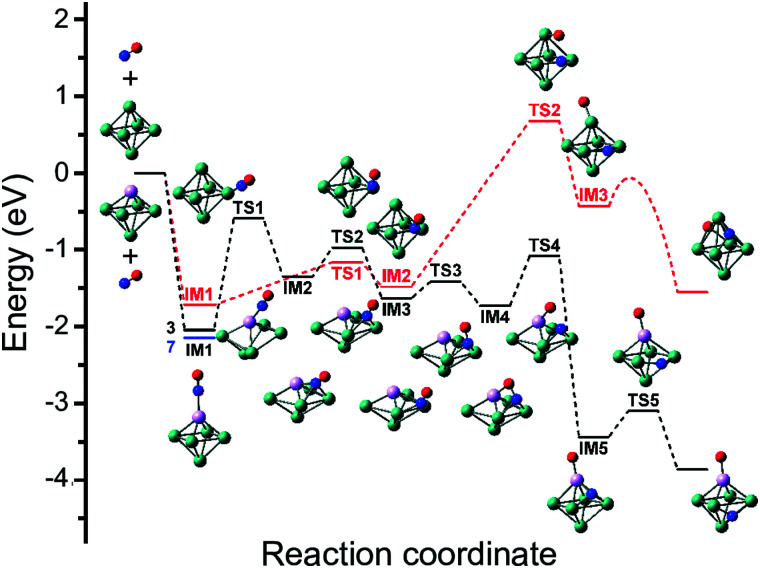
Potential energy surface describing the adsorption and subsequent dissociation of NO on Rh_6_^+^ (red, nonet) and Rh_5_Ta^+^ (black, triplet), calculated at the B3LYP/sdd/aug-cc-pVDZ level. IM indicates intermediate state, TS transition state. For IM1 involving Rh_5_Ta^+^, the lower-lying septet minimum is indicated with a blue line. Adapted with permission from ref. 108. Copyright (2019) American Chemical Society.

Although the dissociative and molecular forms are nearly isoenergetic, the molecularly adsorbed form is clearly dominant for Rh_6_^+^(NO).^91^ The different formation ratios are interpreted as due to the activation barrier between the molecularly and dissociatively adsorbed forms. A reaction pathway for NO adsorption and dissociation on Rh_6_^+^ is shown in red in Fig. 9, where NO first adsorbs on an on-top site of the Rh_6_^+^ cluster *via* the N atom. For NO to dissociate, the system needs to cross a transition state at +0.68 eV with respect to the reactants. After this transition state, a dissociative minimum is reached that is almost isoenergetic with the entrance product where N and O atoms are adsorbed separately on hollow sites. This high activation barrier prevents NO from dissociating. The barrier is likely related to the high dissociation energy of free NO (NO → N + O, Δ*E* = 6.5 eV). Although adsorption on Rh_*n*_^+^ stabilizes the dissociation product and transition state, a rather large barrier remains. Barriers could be lower on other spin surfaces, but both the putative global minimum and the lowest energy molecular isomer found are on the nonet spin surface.

**Fig. 10:**
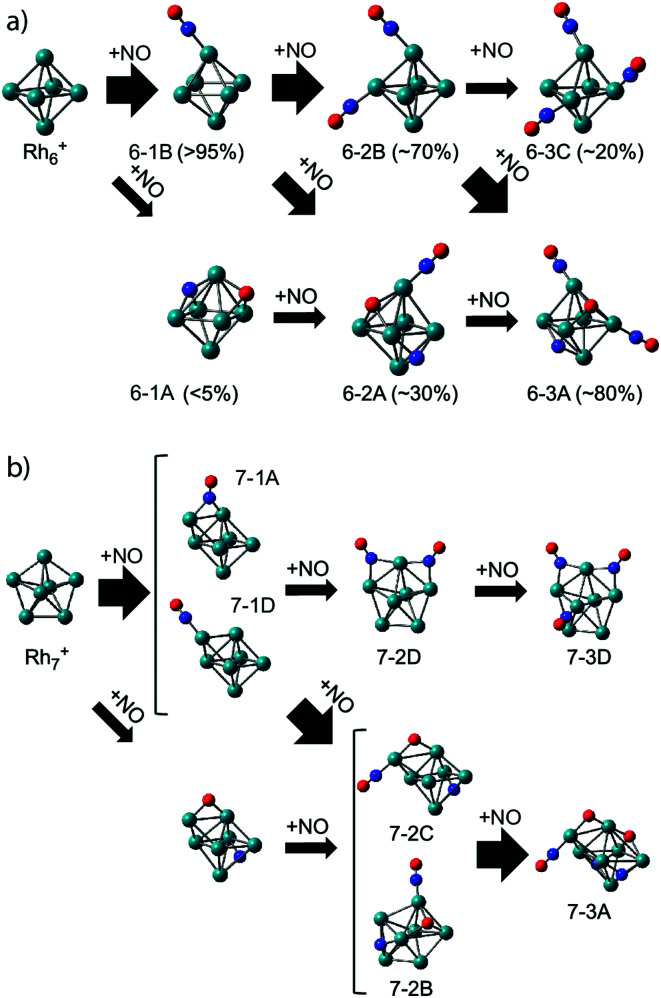
Adsorption pathways of multiple NO molecules onto Rh_6_^+^ (a) and Rh_7_^+^ (b), deduced from experimental population analysis. Reprinted with permission from ref. 109. Copyright (2018) American Chemical Society.

As clusters are known to exhibit significantly changed properties for different cluster sizes, it is of interest to compare the adsorption propensity of NO for Rh_6_^+^ with that for other sizes. Fig. 5 already showed that all sizes *n* = 5–16 have at least a significant fraction of molecularly adsorbed NO. It is worth going into the case of Rh_7_^+^. For Rh_7_^+^(NO), two intense bands are observed at 1600 and 1825 cm^−1^ (Fig. 5) with at least five weaker bands below 700 cm^−1^ (not shown here). NO is concluded to adsorb mainly in a molecular form, with a roughly 4 : 3 ratio for bridge-bound to on-top bound NO (structure 7-1A and 7-1D in Fig. 10, respectively), whereas 5–10% of NO adsorbs dissociatively. Probably most interesting is not so much this throttled dissociation, but that the adsorption may significantly change the cluster structure itself: bare Rh_7_^+^ is a pentagonal bipyramid,^61^ but adsorption of NO leads to a deformation into a capped octahedron for all low-energy structures. This situation is different from Rh_6_^+^, where, even though four isomers are found within 1 eV from the octahedral global minimum,^61,62^ all minima involving molecular adsorption retain the octahedral Rh_6_ framework. Nevertheless, it is clear that the energy released upon adsorption is substantial, and it could lead to significant structural changes, potentially facilitating alternative reaction pathways if the energy is not dissipated.

In line with this, the energy released by adsorption of further NO molecules could enable an NO bound in a molecularly adsorbed entrance channel to dissociate.^62–64^ But what happens when the reaction takes place in the presence of a helium thermalizing buffer gas? The IR-MPD spectra for Rh_6_^+^(NO)_*m*_ (*m* = 2, 3) in Fig. 8 show that these complexes are dominated by molecular adsorption, too.^109^ Of course, now the spectra may have multiple bands in the 1500–2000 cm^−1^ region even if only one isomer exists. An example for this is seen in the calculated spectrum of structure 6-2B. The interpretation of the spectra leads to larger uncertainties in estimating contributions of different isomers, but it is clear that here too contributions of isomers with dissociated NO are found. In contrast to the mass spectrometric work under single-collision conditions,^62–64^ no loss of N_2_ was observed in the mass spectra. This finding reflects the near-thermal nature of the current experiments, where multiple collisions with helium can stabilize intermediate products, and the microcanonical nature of the single-collision experiments. From the IR-MPD spectra and the DFT calculations the picture emerges that the adsorption of extra NO leads to some dissociation, but not much, and that the reduction reaction (formation of N_2_) is out of reach under these conditions. The adsorption steps are shown schematically in Fig. 10. For Rh_6_^+^, after the first NO adsorbs molecularly on an on-top site, the second adsorbs either molecularly on another on-top site or dissociates. Based on the calculated IR absorption strengths and the observed band intensities, we estimate the molecular to dissociative adsorption ratio to be 1 : 0.4. Because we have not calculated further reaction pathways, it is difficult to rationalize this ratio here. Analogously, adsorption of a third NO results in a Rh_6_^+^(NO)_3_ with two molecular NOs adsorbed at on-top sites and one dissociatively adsorbed NO. The results for Rh_6_^+^ parallel those for adsorption of multiple NO onto other cluster sizes: dominance of molecular adsorption and no sign of N_2_ formation was observed for Rh_7_^+^ or Rh_8_^+^.^109^

### Clusters other than pure rhodium

The dissociation of NO on the rhodium surface is the first step of the NO reduction reaction. On the surface of pure Rh clusters, the molecularly adsorbed form is clearly dominant at the temperatures investigated (220 K) because of the activation barrier to dissociation. To understand the specific role of rhodium in these experiments, the adsorption of NO onto clusters of other metals was also examined with IR-MPD spectroscopy.^110–112^

NO adsorption onto Au_*n*_^+^ clusters was studied earlier, but the spectral range probed only allowed observation of NO stretch vibrations.^110^ Despite the observation of odd–even alterations of the vibrational frequencies, no upper limit for the relative population with molecular NO was reported. The change of charge state was evaluated only for Au_4_^−^, with no indication for NO dissociation, even though earlier evidence for NO disproportionation (3NO → N_2_O + NO_2_) was found in mass-spectrometric experiments.^113^

A picture similar to Rh emerged when adsorption onto cationic Ir clusters was studied,^111^ with clear signatures of molecular adsorption. However, molecular adsorption is not as dominant on Ir clusters as it is on Rh clusters: both experimental depletions and relative spectral band strengths pointed at the presence of up to 40–50% of dissociated NO.

To the best of our knowledge, no further spectroscopic studies of pure metal clusters with NO exist. But mixed-metal clusters significantly widen the playing field.^65^ From Fig. 9 it can be inferred that the barrier towards dissociation is formed by a transition state, where the O is peeled off the NO after which it is bound on-top to the Rh_6_^+^ cluster. Thus, if one would want to reduce the barrier, it could be advantageous to stabilize the metal–O bond that is formed after abstraction.

**Fig. 11:**
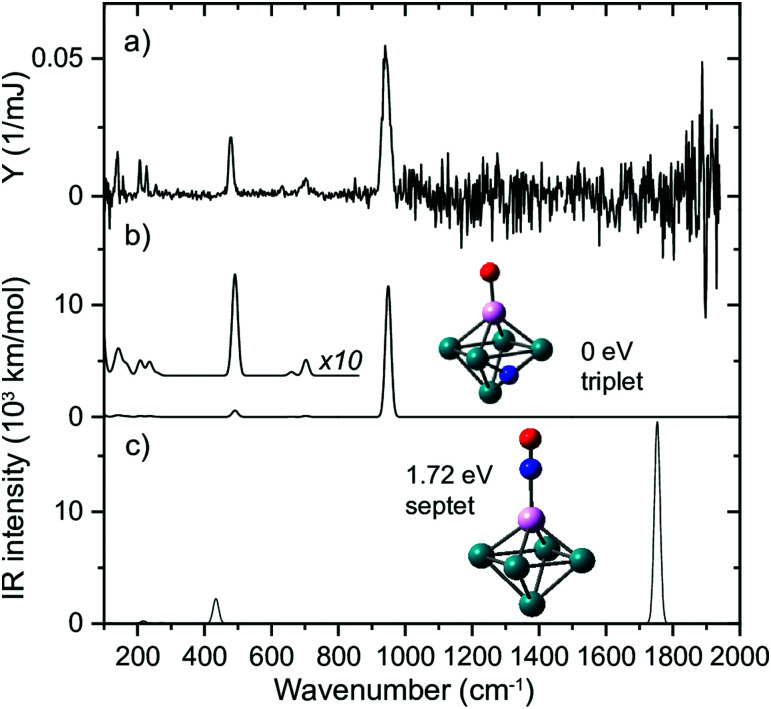
(a) IR MPD spectrum for Rh_5_Ta^+^(NO)Ar, (b + c) harmonic spectra for the shown structures, accompanied by the energy relative to the most stable species, and spin multiplicity. Rh, Ta, N, and O atoms are depicted using green, magenta, blue, and red spheres, respectively. Adapted from ref. 108.

To test this hypothesis, we generated Rh clusters with one Rh atom substituted by a Ta atom, a significantly more oxophilic element than Rh, characterized by bond energies of the neutral diatoms Ta–O (8.2 eV)^114^ compared to Rh–O (4.1 eV).^115^ The resulting spectrum, presented in Fig. 11, shows that adsorption is clearly 100% dissociative, not only for the substituted Rh_6_^+^, TaRh_5_^+^, but for all TaRh_*n*_^+^ (*n* = 2–8).^108^ As sketched above, this is evidenced by lack of any absorption band near 1800 cm^−1^ and the presence of M–O and M–N stretch vibrations. Whereas the adsorption site of the N varied (on a Rh–Rh bridge site for *n* = 2, 3, on a Rh–Rh–Rh hollow site for *n* ≥ 5) the O invariably was found bound on top to the Ta atom.

When reconstructing the reaction pathway for NO dissociation over TaRh_5_^+^ (Fig. 9) it was found that the activation barrier is indeed reduced by more than 1.5 eV, making the barrier exothermic by 1 eV with respect to the reactants. It is well-conceivable that over Rh_6_^+^ a more favorable reaction path involving surface crossings exists, just as for Rh_5_Ta^+^ (where only the triple surface is considered, even though the lowest-energy structure for molecular adsorption is septet, see IM1 in Fig. 9). However, it can hardly be expected that the barrier reduction of more than 1.5 eV will be changed substantially.

**Fig. 12:**
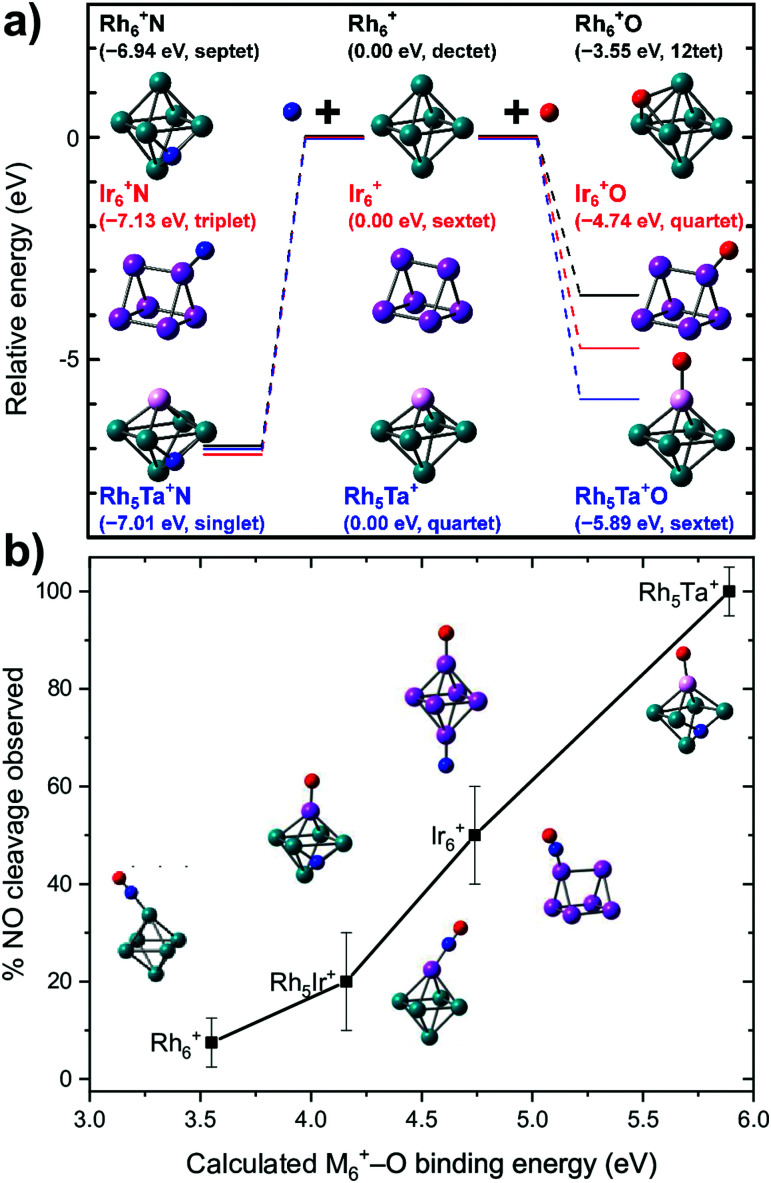
(a) Calculated binding energies of N and O atoms to clusters of various elemental compositions for the site experimentally assigned; (b) propensity for dissociation as a function of the calculated cluster–O binding energy. Top panel: Reprinted with permission from ref. 111 Copyright (2020) American Chemical Society.

What is probably most striking is that the substitution did not lead to large geometric changes: the cluster is still (near-)octahedral, and the abstraction reaction has almost the identical pattern, with the NO attached *via* the N on a bridging Rh–Rh site prior to abstraction, and migrating to a Rh–Rh–Rh hollow site afterwards, while the O is transferred to a Ta rather than a Rh apex. The paths then diverge, as the O prefers a hollow site on Rh_6_^+^, whereas it remains bound to the Ta in the substituted cluster. Obviously, doping an NO reduction catalyst with such an oxophilic element as Ta will not provide a very good catalyst: the O will be so strongly bound that the catalyst will be readily poisoned. However, the manipulation of catalyst material at the single atom level allows to understand the mechanism that drives the NO dissociation. In Fig. 12, the NO dissociation propensity over several six-atomic cluster compositions, is plotted as a function of the highest calculated binding energy of atomic O to the cluster. Here, we see a correlation between the two quantities.^111,116^ This finding indicates that to be able to predict the NO dissociation efficiency, one should evaluate the binding energy of atomic oxygen to the material under debate. This forms a crucial refinement of work by Sakaki and coworkers, who conclude that dissociation is preferred by metals with strong N- and O-affinities (Ru_*n*_ and Rh_*n*_ in particular).^66^ Here, we conclude that the affinity for atomic N, which typically is larger than the affinity for atomic O, facilitates the initial adsorption, whereas it is the O-affinity that governs dissociation efficiency.

Hence, a targeted elemental tailoring of Rh catalyst material is possible by substituting oxophilic elements. Further studies of doped Rh cluster systems to investigate such and other effects, eventually aimed at elucidating a full catalytic NO reduction cycle, are under way.

### Reduction of NO at high temperatures

From the spectroscopy we thus learn that at lower temperatures, NO predominantly adsorbs molecularly onto Rh_*n*_^+^ clusters. Although our spectroscopic studies were limited to the adsorption of three NO molecules, it appears not an unreasonable assumption that this is also the case for larger numbers of NO molecules. The potential dissociation of NO over Rh_*n*_^+^ clusters can also be studied from a different perspective, namely by registering the temperature dependent evaporation of ligands from a Rh_*n*_^+^N_*k*_O_*k*_ species. Although such an experiment gives no conclusive evidence for structure, the appearance of specific loss channels can evidence the reduction of NO, and the temperatures at which this loss has an onset are indicative for the reaction energetics.

The thermally driven evaporation of ligands off Rh_*n*_^+^N_*k*_O_*k*_ was studied using thermal desorption spectrometry.^104,117^ In the He-filled copper extension tube following the reaction gas cell, the clusters reach a thermal equilibrium with the tube wall due to collisions with surrounding He atoms. The NO reduction, which was probed by mass spectrometry, was driven by heat at high temperature. Rh_*n*_^+^N_*k*_O_*k*_, prepared at room temperature, was found below 500 K to only desorb NO molecules sequentially, following

10 Rh_*n*_^+^N_*k*_O_*k*_ → Rh_*n*_^+^N_*k*−1_O_*k*−1_ + NO

11 Rh_*n*_^+^N_*k*−1_O_*k*−1_ → Rh_*n*_^+^N_*k*−2_O_*k*−2_ + NO

which indicates that there are either intact NO molecules in Rh_*n*_^+^N_*k*_O_*k*_ relatively weakly adsorbed on Rh_*n*_^+^, or that they are dissociatively bound, but the back reaction

12 N(a) + O(a) → NO(a) → NO(g)

is preferred over NO reduction and subsequent desorption of N_2_ or O_2_. Further desorption proceeds at higher temperatures (500–1000 K), where only NO is released from the smaller clusters, Rh_*n*_^+^ (*n* ≤ 6).

**Fig. 13:**
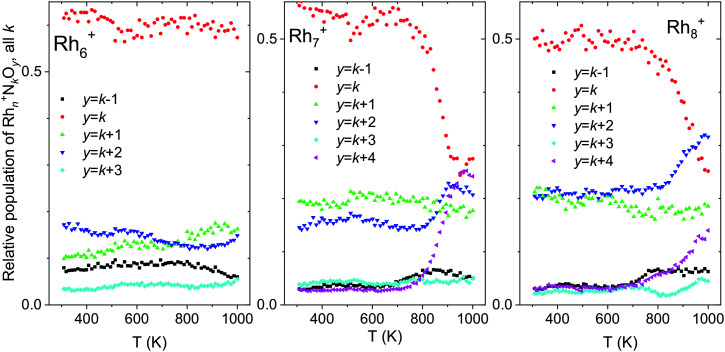
Temperature desorption spectra of Rh_*n*_^+^N_*k*_O_*k*−*m*_ (*n* = 6–8) clusters for *y* = *k* − 1, *k*, *k* + 1,…, *k* + 4, summed over all *k*. Data from ref. 118.

For larger clusters, not only thermal desorption of NO was observed at elevated temperatures, but also N_2_ release. Fig. 13 shows the total intensities of Rh_*n*_^+^N_*k*_O_*y*_, summed for all *k* and with *y* = *k* − 1, *k*, *k* + 1,…, *k* + 4, as a function of temperature. This way of presenting the data does not show the thermal NO desorption, but focuses on loss of other fragments. Rh_*n*_^+^N_*k*_O_*k*+2_ and Rh_*n*_^+^N_*k*_O_*k*+4_, for instance, were presumably formed by the reduction of NO and the subsequent release of N_2_ according to:

13 Rh_*n*_^+^N_*k*+2_O_*k*+2_ → Rh_*n*_^+^N_*k*_O_*k*+2_ + N_2_

14 Rh_*n*_^+^N_*k*+2_O_*k*+4_ → Rh_*n*_^+^N_*k*_O_*k*+4_ + N_2_,

An increase in the total intensities of both Rh_*n*_^+^N_*k*_O_*k*+2_ and Rh_*n*_^+^N_*k*_O_*k*+4_ therefore corresponds to an enhanced reactivity in reactions (13) and (14). In contrast, the formation of Rh_*n*_^+^N_*k*_O_*k*+1_ is interpreted as the release of N_2_O from Rh_*n*_^+^N_*k*+2_O_*k*+2_, while the formation of Rh_*n*_^+^N_*k*+1_O_*k*_ likely results from NO_2_ release from Rh_*n*_^+^N_*k*+2_O_*k*+2_.

15 Rh_*n*_^+^N_*k*+2_O_*k*+2_ → Rh_*n*_^+^N_*k*_O_*k*+1_ + N_2_O

16 Rh_*n*_^+^N_*k*+2_O_*k*+2_ → Rh_*n*_^+^N_*k*+1_O_*k*_ + NO_2_

The traces for *y* = *k* − 1 and *y* = *k* + 1 change only slightly, suggesting that neither NO_2_ nor N_2_O were released in significant amounts, even at higher temperatures. The story is different for *y* = *k* + 2, *k* + 4: for larger clusters (*n* ≥ 7), a clear increase in oxygen-rich clusters can be seen at high temperatures (>800 K). In particular for *n* = 8 (panel c), Rh_8_^+^N_1_O_3_ and Rh_8_^+^O_4_ are produced above 800 K, indicating N_2_ desorption following:

17 Rh_8_^+^N_3_O_3_ → Rh_8_^+^NO_3_ + N_2_

18 Rh_8_^+^N_2_O_4_ → Rh_8_^+^O_4_ + N_2_

These findings are consistent with the spectroscopy: only at higher temperatures NO reduction occurs. The observation that NO reduction occurs for Rh_7_^+^ while it is unclear for Rh_6_^+^ seems consistent with the assignment of the spectrum of Rh_7_^+^(NO)_3_ (not shown here) to a structure with two dissociatively bound NO molecules,^109^ and for Rh_6_^+^(NO)_3_ (Fig. 8) with only one dissociatively bound NO: if two NO molecules are already dissociated at room temperature, the N atoms can presumably more freely migrate over the surface, and at 700 K sufficiently so that they can encounter, form N_2_, and desorb. On Rh_6_^+^, a second NO bond must still rupture, likely requiring a higher activation energy than that for surface mobility.

## Conclusions and future directions

The current work set out to sketch how gas-phase studies can contribute to a fundamental understanding of interactions that play a role in heterogeneous catalysis, in this case the catalytic reduction of NO. Although a direct correspondence with catalytic processes is far from trivial, for instance due to the much higher pressures at which they proceed, the control and level of detail which is attainable in the gas-phase exceeds what can currently be achieved in *operando* studies. Combining experimental results from such well-defined test systems with quantum chemical calculations, offers the possibility to test design concepts for future catalyst materials, as well as providing benchmark data for the development of computational methods describing the catalyst-NO interaction under more realistic conditions.

The current work can be extended along several dimensions. First, there is of course the material dimension, by widening the scope of elements. Here, one can think of other combinations of elements, where apart from NO cleavage efficiency, surface mobility of atomic N and O can be a consideration. Another axis along which this work can be pursued is temperature, where first experiments aimed at combining the TPD experiments with IR structural characterization are currently in preparation in our laboratories. These reactions can also be studied at elevated pressures (∼0.1 mbar) in radio-frequency ion traps as the gas-phase equivalent of chemical reactors, where cluster ions can be reacted under very-well defined experimental conditions, such as partial pressures and temperatures, allowing the controlled driving of reactions, and the recording of temperature dependent reaction kinetics.^52^ When combined with IR spectroscopic characterization of (intermediate) products formed in the ion trap, such kinetics experiments would allow for a full description of the reaction path. Such a complete picture is necessary for understanding potential catalytic action on the scale of clusters, and provides invaluable information needed for the rational design of future catalysts. Further directions could include the resonant heating of cluster-(NO)_*x*_ products through IR absorption. Such heating has been shown to lead to reactions forming other products that could be identified through mass-spectrometry. An example for this is the IR induced dissociation of N_2_O adsorbed on Rh_6_^+^, followed by N_2_ release.^118,119^ Possible promotor effects of the substrate on which catalyst particles are deposited could even be mimicked using for instance fullerenes, as was recently shown for C_60_V^+^ assisted photocatalytic water splitting.^120^ An intriguing direction is the use of picosecond IR pulses to initiate the reaction, and study the time-resolved dynamics of such a reaction. Such a study would complete the characterization of the reactive PES, and is now actively being pursued in our laboratory.

## Conflicts of interest

There are no conflicts to declare.

## Supplementary Material
